# Enhancing sexual health and empowerment among migrant women sex workers: a community health worker-led intervention in Marseille, France

**DOI:** 10.3389/fpubh.2024.1359363

**Published:** 2024-03-27

**Authors:** Emilie Mosnier, Maxime Hoyer, Fernanda Artigas, Hippolyte Regnault, Elodie Richard, David Michels, Marine Mosnier, Grâce Inegbeze, Manuela Salcedo Robledo, Bruno Spire, Stéphanie Vandentorren, Marc Lescaudron, Carole Eldin, Perrine Roux

**Affiliations:** ^1^Aix Marseille Univ, INSERM, IRD, SESSTIM, Sciences Economiques and Sociales de la Santé and Traitement de l’Information Médicale, Aix Marseille Institute of Public Health ISSPAM, Marseille, France; ^2^ANRS-MIE, University of Health Sciences, Phnom Penh, Cambodia; ^3^Prospective Coopération NGO, Marseille, France; ^4^PHAReS, Centre INSERM U1218, Bordeaux Population Health, Université de Bordeaux, Bordeaux, France; ^5^Association AIDES, Pantin, France; ^6^Laboratoire de Recherche Communautaire, Coalition PLUS, Pantin, France; ^7^Unité des Virus Emergents (UVE), Aix-Marseille Université, IRD 190 INSERM 1207 EFS-IRBA, Marseille, France

**Keywords:** community health interventions, women sex workers, migrants, cohort, community-based participatory research, health promotion, community health workers

## Abstract

**Introduction:**

Given the high infection rate of sexually transmitted infections (STI) among migrant women sex workers (WSWs), it is necessary to understand how to improve prevention, information and care for this vulnerable population. Community health workers (CHWs), by linking community to health services, are positioned to improve health outcomes in migrant communities. This article aims to describe a pilot innovative intervention performed by CHWs to improve sexual health in migrant WSWs.

**Methods:**

This one-year intervention study used a respondent-driven sampling (RDS) to recruit a representative cohort of migrant WSWs in Marseille, France. Four CHWs were recruited from different communities and participated in all stages of the research. They performed individual and group interventions of prevention, support in care and empowerment. Data on participant characteristics, type of intervention and adherence to the intervention were reported via questionnaires given to participants. Simultaneously, semi-structured interviews and informal interviews of migrant WSW, CHWs and care providers were carried out.

**Results:**

A total of 132 migrant WSWs were included in the cohort. Very few of them knew about PrEP (12%) or already used HIV post-exposure treatment (9%). Migrant WSWs were often victims of rape or racism, 15 and 21%, respectively. In two-thirds of cases the level of health literacy was low. Participants suffered from a combination of vulnerability factors: difficulties with access to social rights, food or housing. Only 13% reported having benefited from medical follow-up or assistance by an NGO in the 3 months prior to the program. By 3 months, more than one third of the participants had been tested for HIV (35%) and 63% knew about PrEP. A total retention rate of 70% was reported in the cohort after 6 months.

**Conclusion:**

CHWs enabled to improve care access for migrant WSWs by improving the collaboration between care and social actors at a local level. Through these “bring-back-to” interventions for this hard-to-reach population, CHWs enabled an optimization of the care pathway. Our results also highlight the importance of a population-based approach for individual and group support of empowerment interventions in order to strengthen their capacity for action.

## 1 Introduction

In Europe, the epidemic of HIV and sexually transmitted infections (STI) remains particularly active among migrants, who, along with men who have sex with men (MSM), are the populations who are the most vulnerable to these infections (1). Migrants, particularly those from sub-Saharan Africa, represent a significant proportion of people living with HIV in France (PLHIV). Although prevalence in their country of origin is high, a large proportion of these people contract HIV in France due to poor living conditions (2). Moreover, foreign-born PLHIV are also at risk of late detection (3). Finally, foreign-born PLHIV are characterized by a high proportion of women because they combine intersectional risks such as gender inequality, racism, insecurity and violence (4). All these data underline the importance of specific, optimized screening and prevention strategies.

Despite this context, there is a poor perception of risk and consequently little demand for PrEP among migrants (5). The main difficulties encountered by migrant women are unfamiliarity with prevention tools, difficulties in follow-up and in access to social rights. Other specificities that are insufficiently taken into account, such as problems related to contraception, pregnancy, relationships with partners, etc. require holistic approaches (5).

Woman sex workers (WSWs) also represent one of the key populations for controlling the HIV epidemic, due to them having a combination of social, economic and cultural risk factors (6, 7). Several approaches have been tested to reduce HIV incidence in this population. Usually, access to condoms is at the heart of HIV prevention measures. However, these prevention methods are only partially effective (8, 9). In fact, as has been demonstrated, WSWs are not necessarily in a position to impose this type of prevention, particularly for their regular partners (10). WSWs are also more frequently victims of violence which makes the use of this prevention tool difficult (10, 11).

The ANRS-PARCOURS study showed that migrants experience a long period of insecurity upon arrival in France (2). In the PARCOURS study, factors linked to the likelihood of migrant women resorting to transactional relationships included the lack of housing and of residence permits (12). Thus, the factors of precariousness (housing, food, right to work, etc.) among migrant women are at the origin of SW activity. While community-based risk reduction programs for MSM have been successful, particularly in terms of levels of knowledge and access to PrEP, the same cannot be said for migrant populations (13).

All these factors point to the growing importance of community health programs in public health strategies. The development of these programs and the need for health mediation and Community Health Workers (CHWs) are now widely recognized, both nationally and internationally (14, 15). Community health is thus an integral part of public health, representing a strategy within health promotion approaches. Its specific feature is that it is population-based rather than individual-based, and promotes and implements a holistic vision of health on a fine territorial level. In practice, benefits are expected at individual, community and institutional levels. The power of these programs lies in the fact that they aim to empower people and communities. However, evaluation and scaling up of community health initiatives are hampered by the length of time needed to measure their effects, and by the fact that they are often adapted to a specific context and not easily replicated or synchronized (16). In order to understand better the different levels at which community health interventions can act, detailed intervention descriptions are needed.

This article aims to describe, via a cohort conducted as part of a pilot intervention to improve sexual health in migrant WSWs, the characteristics of this population and the actions implemented by CHWs.

## 2 Materials and methods

### 2.1 Study design

This cohort, with a mixed method descriptive analysis, is an ancillary study of the FASSETS program (Favoriser l’Accés à la Santé SExuelle des Travailleuses du Sexe). The program protocol has already been published (17). The cohort was set up using Respondent-Driven Sampling (RDS). A 6-month follow-up was set up with a quantitative and qualitative evaluation. CHWs carried out the interventions throughout the follow-up period.

### 2.2 Community-based participatory research

The FASSETS study is a community-based participatory study. Community-based participatory research is an approach to research that involves a collective, reflective and systematic study in which the researcher and community stakeholders are involved in all stages of the research process with the aim of improving practices (18). This approach promotes shared control over individual and collective health and social conditions (19, 20). The FASSETS project was initially developed around two community-based non-governmental organizations (NGOs): AIDES and The Truth, which provide community-based sexual and reproductive health interventions for migrant sex workers. These NGOs include CHWs from the communities involved in the research. In line with the principles of community-based participatory research, this study involved the creation of partnerships, regular exchanges between partners and community organizations/groups, and the sharing of experiences between researchers, CHWs and the community of migrant WSWs.

### 2.3 Population, study area and recruitment

#### 2.3.1 Study area

The study sites included all of the medical and social care sites, including outreach activities, for migrant WSWs in Marseille, as well as the inclusion and follow-up site specific to the FASSETS study.

#### 2.3.2 Population study

The study population included: (i) the 4 CHWs from Nigeria, Eastern Europe, Brazil and North Africa (two of the four are peer workers), (ii) the partners: health institutions and organizations involved in sexual health care in Marseille (Planning familial, Amicale du Nid, CEGIDD, AIDES, Autres Regards, COREVIH, ARS etc.) and (iii) the migrant WSWs accompanied by the FASSETS mediators.

#### 2.3.3 Recruitment of migrant WSW

As migrant WSWs are a hidden population, the FASSETS study used the Respondent-Driven Sampling method to obtain a cohort as representative as possible of the different migrant WSW communities, whether or not they were already in the care system (21). There were 10 seeds in total, of different ages and belonging to cis or transgender women’s communities, who were from sub-Saharan Africa, North Africa, South America and Eastern Europe communities.

#### 2.3.4 Inclusion criteria

The criteria for inclusion in the FASSETS cohort were: (i) cis or transgender women over 18 years of age, (ii) having provided sexual services in exchange for a service or monetary compensation during the last 12 months, (iii) born abroad, (iv) working, living or regularly passing through Marseille and (v) having signed a free and informed consent to take part in this study. Non-mastery of the French language was not a criterion for exclusion. Questionnaires and follow-up were conducted by CHWs in the participants’ native language.

### 2.4 Data collection

The FASSETS CHWs were previously trained in the survey technique by researchers and infectious disease physicians. They collected quantitative data from the migrant WSWs using a standardized tablet questionnaire. The interview method was face-to-face with CHWs in the participant’s native language for the first questionnaire and the two following ones. Additional follow-up by CHWs could be face-to-face, by telephone or via social networks, depending on needs. They also listed all the actions carried out and how they were implemented. Members of the project team worked with a team of sociologists to collect qualitative data from the WSWs. Data on partners was collected by questionnaires and focus groups.

Variables were collected through standardized questionnaires and included demographic characteristics, sex work characteristics, behavioral characteristics (alcohol use, drug use), medical history, level of health literacy and needs in sexual health. Perceived stress was measured using the Perceived Stress Scale (PSS) (22). A committee of experts from outside the study reviewed the questionnaire. Questions and variables were standardized on previous PARCOURS studies, on precariousness scales and on the conceptual framework for describing CHW activities previously validated in the literature (12, 23, 24).

For this ancillary study, only data collected between April (the start of the study) and November 2022 were analyzed.

### 2.5 Analysis

#### 2.5.1 Quantitative analysis

Categorical variables were compared using Fisher’s exact test. Means were compared using Student’s t-test. The association between lost to follow-up and explanatory variables were assessed using univariate analysis. All *p* < 0.05 were considered statistically significant. Statistical analysis was carried out using R software (R Foundation for Statistical Computing, 3.3.1-Studio).

#### 2.5.2 Qualitative analysis

An inductive approach was used, particularly adapted to exploratory research. Questions were focused on the needs and representation of sexual health and care among migrant WSWs. Health care providers, CHWs and migrant WSWs were interviewed. Migrant WSWs were encouraged to share specific experiences such as stories from their own lives to generate conversation. A revision workshop was organized where researchers provided a summary of the data collected to the participants in order to get their feedback and confirmation of results. The interviews and notes of these exchanges were transcribed and were subjected to manual thematic coding. A total of 13 semi-structured and 12 informal interviews were performed.

Qualitative and quantitative data were collected and analyzed, respectively, and simultaneously, followed by a triangulation of qualitative and quantitative data to interpret the results.

### 2.6 Ethics

The protocol received approval from French Ethics Committee in October 2021 (no. 2021—A01746-35).

## 3 Results

### 3.1 Characteristics of the migrant WSW participants

A total of 132 participants were included in the study. Table 1 provides population estimates of socio-demographic characteristics. More than half could not write (*n* = 85/132, 65%) and the level of health literacy was low (Table 1).

**Table 1:** Socio-demographic, social, disease and needs of health service variables and indexes.

	Variables	Values	%	*N* = 132
Socio-demographics	Age (mean ± SD; years)	35 ± 13; (22–82)	100	132
	Gender	Cis	95	125
		Trans	5	7
	Native community	Sub-Saharan Africa	69	91
		North Africa	18	24
		Eastern Europe	8	11
		Other	5	6
	Family status in France	Alone	57	76
		With family or friends	42	56
Social	Homeless^1^	Yes	64	85
		No	35	47
	Food insecurity	Yes	35	47
		No	64	85
	No social security coverage	Yes	72	95
		No	28	37
	No resident permit	Yes	18	24
		No	82	108
Health literacy	I can follow the instructions of health professionals	Yes	39	51
		No	61	79
	I can read and understand health information	Yes	23	30
		No	77	100
	I can read and understand instructions on taking medication	Yes	28	37
		No	72	93
	I can write	Yes	37	49
No		63	83
Disease	Medical history of STI	Yes	10	13
		No	90	119
	HIV positive status	Yes	1	1
		No or no answer	99	131
	Drug use^2^	Yes	5	7
		No	95	127
	Alcohol use	Yes	14	19
		No or less than 3 drinks per week	86	115
	Victim of violence^3^ during the last 12 months	Yes	15	20
		No	85	111
	Victim of racism during the last 12 months	Yes	21	28
		No	78	103
Needs for health service	Followed by a health institution for their sexual health	Yes	14	18
		No	86	114
	History of HIV testing	Yes	81	109
		No	19	25
	Knows about PrEP^4^	Yes	12	16
		No	88	117
	Already used Post-Exposure treatment for HIV	Yes	2	3
		No	98	123

^1^According to ETHOS definition (living in the street, emergency shelters, squat or slum) (23). ^2^Other drugs than alcohol or tobacco. ^3^Rape or physical violence. ^4^HIV pre-exposure treatment.

The majority of migrant WSWs were socially very vulnerable. In over half of the cases they were homeless, and they reported food insecurity or having no social security coverage or resident permit (Table 1).

During the previous 12 months a significant number of participants reported having suffered from violence and racism (Table 1).

The majority of participants (87%, *n* = 114) said that they had had no contact with NGOs or institutional care structures for their sexual health within the last 3 months.

Most Migrant WSW had children (87%, *n* = 115) with a mean of 2.12 children. They had been in France for an average of 7.8 years (SD = 10.1).

A total of 56% (*n* = 56) of participants assessed had a low level of stress, 32% (*n* = 30) a moderate level, and 12% (*n* = 11) a high level of perceived stress according the PSS.

**Figure 1:**
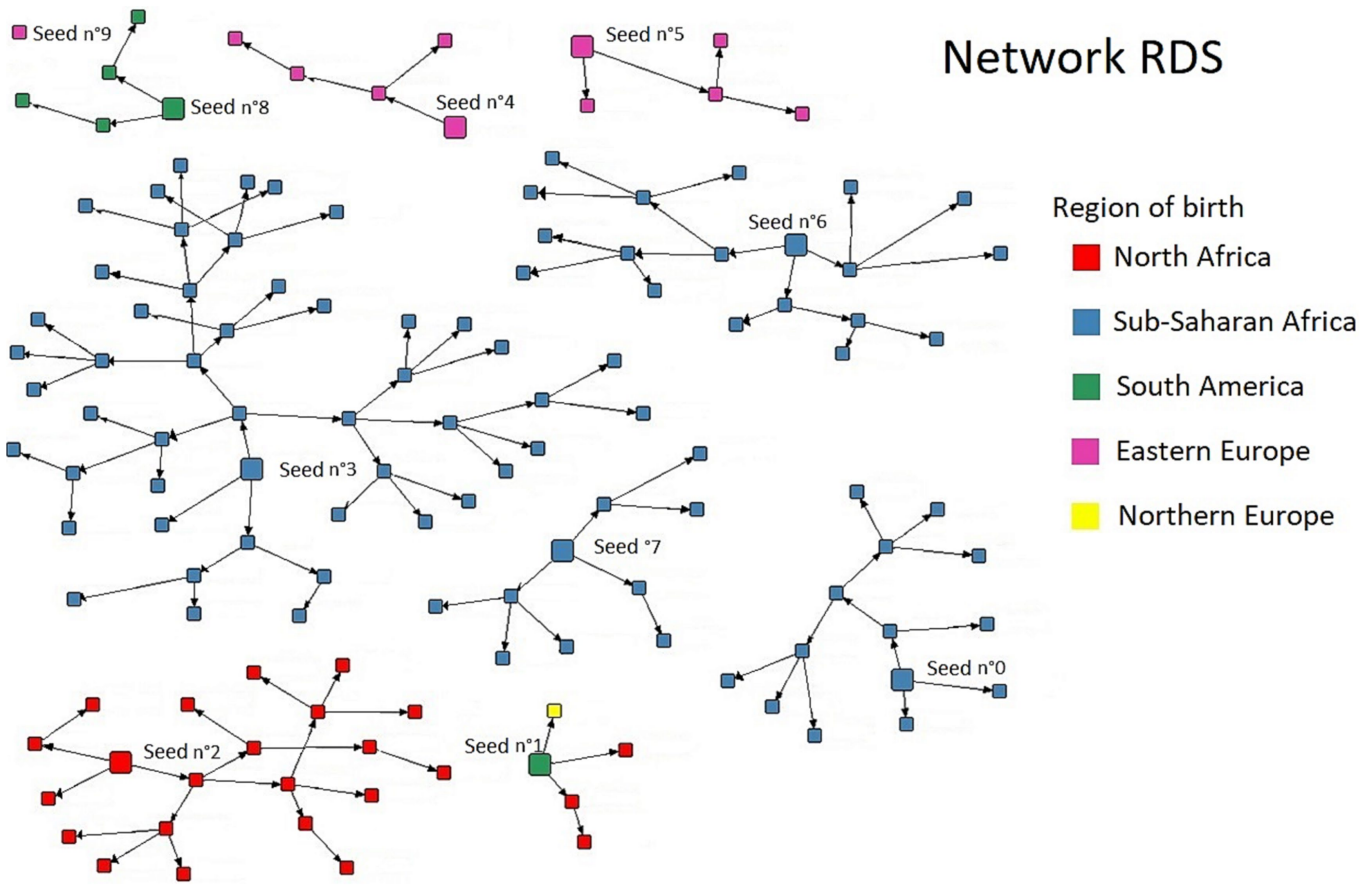
Result of the respondent-driven sampling in migrant WSWs.

The RDS reported a near-perfect homophily of the communities network (Figure 1). A total of 10 seeds were used. The RDS was stopped at the third wave for reasons of human resource capacity (CHWs) to monitor the recruited cohort correctly.

### 3.2 FASSETS activities of CHWs

A total of 4 CHWs were recruited from the various regions of origin of the migrant WSWs. Two of them were peer workers. All were women and one of them was transgender. CHWs activities are very varied, an example of a CHW’s activities is shown in Table 2.

**Table 2:** Example of the activity of one community health worker from April 2022 to November 2022.

		%	*N*
Person/place requiring a community health worker. *N* = 14	Health care center	30	5
	Welfare institutions (housing, food)	27	4
	Migrant women sex workers	43	6
Type of action. *N* = 177	Linkage to care	37	66
	Information and support	51	91
	Guidance	6.8	12
	Other	4.5	8
Referral to. *N* = 13	Health care center	31	4
	Welfare institutions (housing, food)	54	7
	Other	15	2

**Figure 2:**
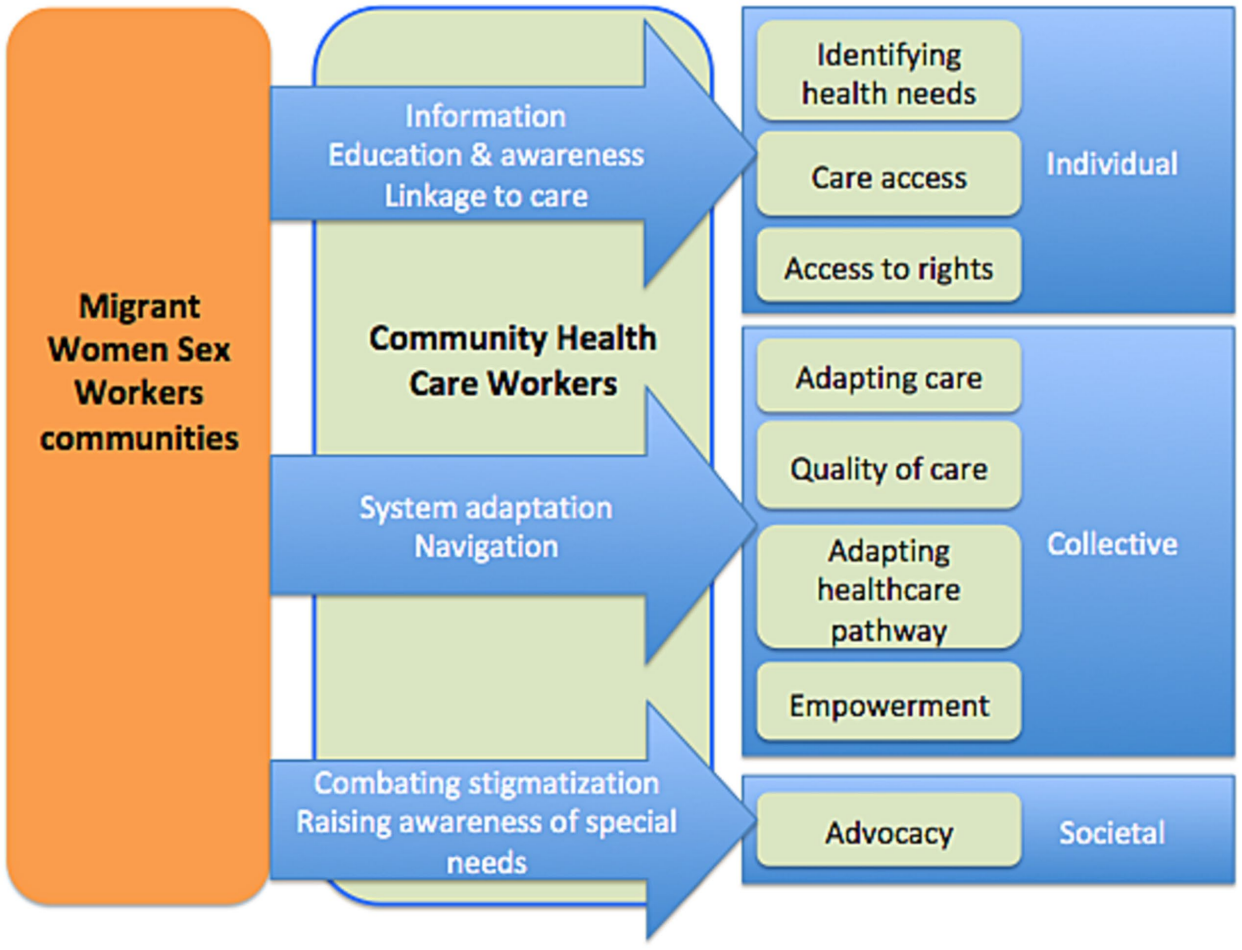
Type and level of community health workers health actions with migrant women sex workers.

**Figure 3:**
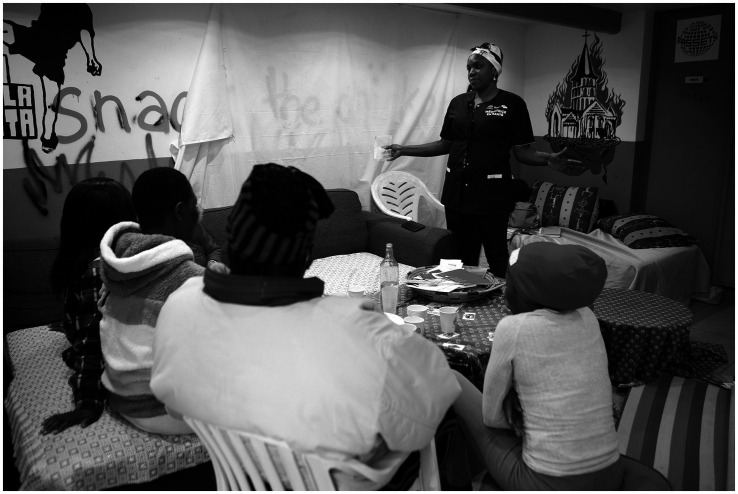
Example of an empowerment group for Nigerian women on sexual health and information about PrEP.

The actions implemented by CHWs to improve the sexual health of migrant WSWs take place at different levels (Table 3; Figure 2). At an individual level, it is necessary to perceive the person’s health needs (all the more difficult in the presence of discrimination or a competitiveness of needs, notably here with access to social rights, food and family needs), then to improve levels of knowledge in sexual health and facilitate access to care. Taking into account past experiences, vicarious experiences, medical history and perceptions also play a role in the migrant WSWs’ self-efficacy and capacity to act. The CHWs also work on a collective and social level to gain recognition of the participants’ specificities, notably when setting up care pathways dedicated to women (adaptation of the care system) or their advocacy actions (Figures 2, 3).

**Table 3:** Description of actions carried out among and with migrant women sex workers.

Level of action	Stakeholders	Description of actions	Goals
Individual	migrant WSWs, CHWs	Linkage to care	Access to care, increase prevention and screening
	migrant WSWs, CHWs	Information/Support	Improve knowledge, self-esteem
	migrant WSWs, CHWs	Access to rights, housing, food aid, French courses and schooling for their children	Improving holistic approach to care
Group	migrant WSWs, CHWs Mainly sub-Saharan community	Information during a group meal on sexual harm reduction	Improve knowledge of STIs and how to prevent them
	migrant WSWs, CHWs Mainly North African Community	Seaside outing	Fighting stigma and boosting self-esteem
	migrant WSWs, CHWs	Collective HIV screening and support	Improve screening rates and knowledge of care pathways
	migrant WSWs, CHWs Mainly Transgender community and sub-Saharan community	Tiktok video with dance	Improve self-esteem, talk positively about sexual health and knowledge of the care network
	migrant WSWs, CHWs Mainly sub-Saharan community	Photo workshop and camera giveaway	Improve self-esteem, talk positively about sexual health
	migrant WSWs, CHWs Mainly Transgender	Information about drug interactions with hormone therapy and health care pathway for hormone therapy	Improve uptake of PrEP in the transgender women group
	migrant WSWs, CHWs	Workshop on domestic violence	Improve knowledge of gender-based violence and how to prevent it
	migrant WSWs, CHWs	Workshop on access to rights	Improve access to rights
	migrant WSWs, CHWs Mainly sub-Saharan community	Workshop on female genital mutilation	Improve knowledge of female genital mutilation and how to prevent it and take care of the women
Institutions and policy makers	migrant WSWs, CHWs Health care providers, researchers, community based organizations	Workshop with the first dissemination of the FASSETS study results	Improve: Specific needs of migrant WSWs Healthcare pathway
	CHWs and researchers	Contribution to regional coordination initiatives	Information on migrant WSWs’ characteristics and public health issues Synergy of actions between networks
	CHWs and researchers	Scientific communication (oral communication, articles)	Information on migrant WSWs’ characteristics and public health issues
	CHWs and researchers	Newsletter	Information on migrant WSWs’ characteristics and public health issues

It is clear from these actions (Table 3) that the CHWs enable the needs formulated by the MWSWs to be incorporated into collective actions and have an impact on institutions (Figure 2).

CHWs take into account the complex intersections between structural barriers affecting migrant WSWs and their gender, education, race, and socio-economic status. Based on the needs expressed by migrant WSWs, CHWs not only optimize access to care but also impact it by encouraging adaptations (Figure 2).

### 3.3 Qualitative multilevel assessment (migrant WSWs, CHWs and health care providers), different needs according to communities

The results of the interviews showed that cisgender women differ from transgender women (TGW) in their perception of the sexual health risks to which they are exposed, in their knowledge of prevention tools and in the expression of their needs to the medical-social sector.

“Normally South American [trans] girls are mainly looking for access to care, health, help with their papers, it’s stuff… frankly it’s light… when they arrive at the association they know what they’re looking for, what they want… it’s different from Nigerian girls where you have to unpack the whole package to try and find out what they want…” (CHW, sex workers NGO).

For the NGOs, TGW are more receptive to sexual health issues. According to health care workers, migrant TGW feel much more “at risk” of contracting HIV and other STIs than migrant cisgender women.

There are also community-specific characteristics, particularly among Nigerian women, related to the violence and trauma of migration.

“As I was telling you earlier, there's normal prostitution and forced prostitution… and right now we’re taking much more care of Nigerian girls than other WSWs at the association… 70% of our active file I think. As these are girls who have experienced rape, exploitation… they don’t see prostitution as work…” (Health mediator, association for sex workers).

The migrant WSWs interviewed frequently told stories of rape, particularly during their migration pathway. Most of the Nigerian women reported psychosomatic trauma, and said they were not treated for post-traumatic stress disorder due to a lack of English-speaking psychologists, who also refused to treat migrants without documents. Migrant WSWs from North Africa, who have been living in France for longer, reported a particular need to fight stigmatization and build social ties. Eastern European migrant WSWs reported frequent trips back to their home countries to see their families and often their children, limiting their follow-up in care facilities.

Participants, particularly those of sub-Saharan origin, also often mentioned the notion of excision and lack of knowledge of their own body.

“Most don’t know if they have been or not” (Health care worker talking about genital mutilation/cutting).

### 3.4 Impact of community health interventions during follow-up and retention

Upon inclusion, 28% (*n* = 37/132) of migrant WSWs felt that the CHWs could not help them with their sexual health. Moreover, the vast majority of migrant WSWs (69%, *n* = 93/132) provided no help to their peers to improve their sexual health.

At 3 months, 63% (*n* = 60/95) of participants knew about PrEP. A total of 21% of migrant WSWs were referred to care for their sexual health needs. More than one third of participants had been tested for HIV (34%, *n* = 33/95).

At 6 months of follow-up, 45% (*n* = 42/93) of migrant WSWs declared having asked the CHWs for help with their care.

At 3 and 6 months of follow-up by the CHWs, the cohort retention rates were 72 and 70%, respectively.

## 4 Discussion

This study used a community-based recruitment and follow-up method. The main results from the migrant population showed relatively closed networks and community-specific needs. CHWs were able to set up multi-level actions with the migrant WSWs, while adapting to the local healthcare offer and the needs of each community.

The RDS network results of our study showed a significant compartmentalization of the migrant WSWs network. These sub-networks are structured around communities of belonging linked to the migrant WSWs country and geographic region of origin. Indeed, these results show the importance of community mediation actions investing in these sub-networks. Few studies exist about the characteristics of interactions between sex workers (25). Some studies provide some information about exchanges of social support and others resources between partners, clients, peers or co-workers (26, 27), but data is lacking on the organization and quality of the relationships described. Indeed, trust between migrant WSWs and CHWs is a key element in the linkage to care. Here we show that groups are compartmentalized, have different needs and require specific actions. The TGW community seemed, in our program, to have a better understanding of STI prevention issues than cisgender migrant women. One hypothesis is that they benefit more from the LGBTQ+ community network and targeted communication campaigns. TGWs may also benefit from information and support via LGBTQ+ Social Network apps (28). Furthermore, other studies comparing cis and transgender black women have shown that beyond gender, it is access to employment that is linked to prevention practices (29). Unfortunately, we can also confirm, notably in the sub-Saharan community, the high rate of post-traumatic stress disorder linked to the violence experienced during the migration pathway, and its consequences in terms of mental health (30, 31).

CHWs seem to be able to adapt actions to needs at individual and collective levels. They also enable institutions to adapt themselves in terms of care provision or recognition of specific community health needs. This study is exploratory but seems to show an impact even after 3 months on knowledge and screening practices. These results need to be confirmed with longer follow-up times and larger groups.

These results also showed women with a combination of vulnerability factors: lack of housing, multiple forms of violence and food insecurity. This calls for a complex, holistic approach on multiple levels. Indeed, the WHO is increasingly recommending integrated approaches for STI prevention and care (32). Levels of insecurity are high and probably lead to significant risk-taking (2). The participants have to meet their own needs as well as those of their families, and studies have shown that the presence of dependent children exacerbates the need for money and sexual risk-taking (33). There is a competitiveness of needs, with those of the children always taking precedence over those of the mother. Illegal status on the territory, lack of housing, multiple forms of violence and food insecurity mean that migrant WSWs need an approach that prioritizes their urgent needs. Numerous studies and “housing first” programs have shown that, in order to be effective, all preventive and therapeutic actions must first ensure the safety of the beneficiaries (34). Given the complexity of needs, CHWs could help optimize access to local resources, but this would clearly not be sufficient in a context of chronic lack of accommodation and food aid in France for the most vulnerable (35).

Community health work involves information-, education-, and communication-based activities, as well as health system navigation activities, and includes activities to involve professionals, stakeholders and communities. Health mediation is thus both a promising intervention (“one whose effectiveness has not been evaluated by research, but for which a solid normative evaluation induces a presumption of relevant results” (36)) and a complex one to study, in that it combines varied practices that require constant adaptation to a socially shifting context. Our results show that community health interventions with empowerment actions are part of an ecological concept that applies to interactive change at several levels: the individual, the organization and the community (37). Working on mediation issues therefore implies studying not only individual changes, but also changes in the social setting itself. In fact, to our knowledge, with the exception of some pioneering studies (38, 39), no study has been able to demonstrate its effectiveness by taking into account the conditions of the effect of mediation on health, because this evaluation requires integrating all this complexity. In fact, as mentioned above, healthcare mediation practices are multi-faceted, constantly adapting and multi-level (24). They make up an interventional system centered around the individual (40). Following this systemic approach, Richard et al. (20) proposed to map the theoretical conditions of feasibility and success of health mediation to promote the use of health services within a conceptual framework characterizing their potential interrelationships, which it would be interesting to map out in real life.

This study provides community data from a hidden key population, who are without linkage to care. Indeed, migrant WSWs do not receive care in the usual screening centers in the study region (41). Specific information on these communities is required to control the STI epidemic. More and more studies and programs are using RDS to reach these populations and enable specific interventions (42, 43). Furthermore, our findings demonstrated that migrant WSWs were able to recruit others, regardless of the level of education or vulnerability, showing how RDS could reach participants, even those with an illegal status and not followed by the NGOs network, within a short recruitment period. To our knowledge, this is the first RDS study in France on sex workers or on migrants. However, other RDS studies have been successfully carried out in other countries among sex worker populations (44, 45).

Some limitations have to be acknowledged. The study’s findings may lack generalizability due to its specific focus on Marseille and a relatively small sample size. Sampling bias and the absence of a control group limit the ability to establish a causal relationship between community health worker interventions and observed outcomes. The small size of the study population precluded extensive multivariate analysis, especially of subgroups. The short six-month follow-up period may not capture sustained changes over time. Additionally, reliance on self-reported data introduces the potential for recall and social desirability biases. However, this is important exploratory research within a difficult-to-reach population, which will allow us to set up more specific interventional studies.

In conclusion, CHWs can carry out complex and comprehensive interventions with migrant WSWs. This key population has multiple vulnerability factors and requires a holistic approach that takes into account different levels of network: the women’s family (especially their children), their community of origin to which they seem extremely connected, and the local care network to which they have little access despite their substantial needs. Other studies are required, using specific methodologies, to evaluate CHW actions, their acceptability, their cost effectiveness, and their impact on the health of beneficiaries and on public health. Finally, these actions carried out by harm-reduction CHWs should be accompanied by public policy measures to help secure the most vulnerable people, at the very least in terms of protection from stigma, discrimination and violence, and by providing emergency accommodation and food aid.

## Data availability statement

The original contributions presented in the study are included in the article/supplementary materials, further inquiries can be directed to the corresponding author.

## Ethics statement

The studies involving humans were approved by French Ethics Committee in October 2021 (no. 2021—A01746-35). The studies were conducted in accordance with the local legislation and institutional requirements. The participants provided their written informed consent to participate in this study. Written informed consent was obtained from the individual(s) for the publication of any identifiable images or data included in this article.

## Author contributions

EM: Writing – original draft, Writing – review & editing, Conceptualization, Data curation, Formal analysis, Funding acquisition, Investigation, Methodology, Project administration, Resources, Software, Supervision, Validation, Visualization. MH: Conceptualization, Data curation, Formal analysis, Investigation, Methodology, Software, Writing – original draft, Writing – review & editing, Supervision. FA: Conceptualization, Data curation, Funding acquisition, Investigation, Methodology, Project administration, Resources, Validation, Visualization, Writing – original draft, Writing – review & editing. HR: Data curation, Methodology, Validation, Writing – review & editing. ER: Conceptualization, Methodology, Validation, Writing – review & editing. DM: Conceptualization, Methodology, Validation, Writing – review & editing. MM: Conceptualization, Data curation, Funding acquisition, Investigation, Methodology, Project administration, Validation, Writing – review & editing. GI: Data curation, Investigation, Project administration, Validation, Writing – review & editing. MR: Conceptualization, Data curation, Project administration, Writing – review & editing. BS: Conceptualization, Methodology, Supervision, Writing – review & editing. SV: Conceptualization, Investigation, Methodology, Supervision, Writing – review & editing. ML: Data curation, Funding acquisition, Project administration, Resources, Validation, Writing – review & editing. CE: Conceptualization, Formal analysis, Investigation, Methodology, Project administration, Supervision, Writing – review & editing. PR: Writing – original draft, Writing – review & editing.
